# The first study on analysis of the codon usage bias and evolutionary analysis of the glycoprotein envelope E2 gene of seven *Pestiviruses*

**DOI:** 10.14202/vetworld.2022.1857-1868

**Published:** 2022-07-29

**Authors:** Mohammad Shueb, Shashanka K. Prasad, Kuralayanapalya Puttahonnappa Suresh, Uma Bharathi Indrabalan, Mallikarjun S. Beelagi, Chandan Shivamallu, Ekaterina Silina, Victor Stupin, Natalia Manturova, Shiva Prasad Kollur, Bibek Ranjan Shome, Raghu Ram Achar, Sharanagouda S. Patil

**Affiliations:** 1 Department of Biotechnology and Bioinformatics, School of Life Sciences, JSS Academy of Higher Education and Research, Mysuru, Karnataka, India; 2 ICAR-National Institute of Veterinary Epidemiology and Disease Informatics (NIVEDI), Yelahanka, Bengaluru, Karnataka, India; 3 Department of Surgery, Pirogov Russian National Research Medical University, 117997, Moscow, Russia; 4 School of Physical Sciences, Amrita Vishwa Vidyapeetham, Mysuru, India; 5 Division of Biochemistry, School of Life Sciences, JSS Academy of Higher Education and Research, Mysuru, India

**Keywords:** codon usage bias, evolutionary analysis, *Flaviviridae*, glycoprotein E2, India, *Pestivirus*

## Abstract

**Background and Aim:**

*Pestivirus*, a genus of the Flaviviridae family, comprises viruses that affect bovines, sheep, and pigs. Symptoms, including hemorrhagic syndromes, abortion, respiratory complications, and deadly mucosal diseases, are produced in infected animals, which cause huge economic losses to the farmers. Bovine viral diarrhea virus-1, bovine viral diarrhea virus-2, classical swine fever virus, border disease virus, Bungowannah, Hobi-like, and atypical porcine pestivirus belonging to the *Pestivirus* genus were selected for the study. This study aimed to estimate the codon usage bias and the rate of evolution using the glycoprotein E2 gene. Furthermore, codon usage bias analysis was performed using publicly available nucleotide sequences of the E2 gene of all seven *Pestiviruses*. These nucleotide sequences might elucidate the disease epidemiology and facilitate the development of designing better vaccines.

**Materials and Methods:**

Coding sequences of the E2 gene of *Pestiviruses A* (n = 89), *B* (n = 60), *C* (n = 75), *D* (n = 10), *F* (n = 07), *H* (n = 52), and *K* (n = 85) were included in this study. They were analyzed using different methods to estimate the codon usage bias and evolution. In addition, the maximum likelihood and Bayesian methodologies were employed to analyze a molecular dataset of seven *Pestivirus*es using a complete E2 gene region.

**Results:**

The combined analysis of codon usage bias and evolutionary rate analysis revealed that the *Pestivirus*es *A, B, C, D, F, H*, and *K* have a codon usage bias in which mutation and natural selection have played vital roles. Furthermore, while the effective number of codons values revealed a moderate bias, neutrality plots indicated the natural selection in *A, B, F*, and *H* *Pestivirus*es and mutational pressure in *C, D*, and *K* *Pestivirus*es. The correspondence analysis revealed that axis-1 significantly contributes to the synonymous codon usage pattern. In this study, the evolutionary rate of *Pestiviruses* *B*, *H*, and *K* was very high. The most recent common ancestors of all *Pestivirus* lineages are 1997, 1975, 1946, 1990, 2004, 1990, and 1990 for *Pestiviruses A, B, C, D, F, H*, and *K*, respectively. This study confirms that both mutational pressure and natural selection have played a significant role in codon usage bias and evolutionary studies.

**Conclusion:**

This study provides insight into the codon usage bias and evolutionary lineages of *pestiviruses*. It is arguably the first report of such kind. The information provided by the study can be further used to elucidate the respective host adaptation strategies of the viruses. In turn, this information helps study the epidemiology and control methods of *pestiviruses*.

## Introduction

*Pestivirus*, a genus of the *Flaviviridae* family comprising 11 species, infects bovines, sheep, and pigs. They are approximately 50 nm in diameter, exhibiting spherically enveloped geometries. In addition, genomes are linear in structure, being approximately 12 kb in length. The attachment of the viral envelope protein E to host receptors, which mediates clathrin-mediated endocytosis, allows the virus to enter the host cell. Thus, the replication model for positive-stranded RNA viruses was used in this study. The technique of transcription is based on the positive-stranded RNA viral transcription. This process of translation is initiated by a virus. By budding, the virus leaves the host cell. Mammalian hosts are natural hosts, and parental transmission pathways exist [1, 2]. *Pestiviruses* feature a single strand of positive-sense RNA that is approximately 12.5 kb long. Usually, the 3’ end of the genome has no Poly-A. RNA in the genome encodes both structural and nonstructural proteins [3].

Recently, *Pestiviruses* were classified into the following categories, namely, *Pestivirus A*, *Pestivirus B*, *Pestivirus C*, *Pestivirus D*, *Pestivirus F*, *Pestivirus H*, and *Pestivirus K*, which were included in our study. *Pestivirus A*, also known as bovine viral diarrhea virus 1 (BVDV-1), causes bovine viral diarrhea and mucosal disease; *Pestivirus B*, also known as bovine viral diarrhea virus 2 (BVDV-2), causes bovine viral diarrhea and mucosal disease; *Pestivirus C*, often known as classical swine fever virus (CSFV), causes classical swine fever; *Pestivirus D*, often known as border disease virus (BDV), causes border disease; *Pestivirus F* is also known as Bungowannah virus; *Pestivirus H* is also known as Hobi-like pestivirus; and *Pestivirus K* is also known as atypical porcine pestivirus [4].

The codon usage bias is the most favorable element in host-virus evolution. Codon bias is defined as the non-random selection of synonymous codons for each gene or genome. Furthermore, it is organism-specific, where it can be influenced by GC content, gene lengths, and gene expression level. To comprehend the molecular process of expression and the impact of long-term evolution on a genome, it is necessary to investigate the recognition of a diverse pattern of codons with distinct biological consequences. Codon bias is the most preferred and widely used hypothetical analytic tool for analyzing codon usage [5, 6]. The determination of codon usage reflects the aggregate effects of three evolutionary forces, including genetic drift within a sample, natural selection, and mutational pressure. Overall, shuffle in the GC and AT (U) pairs cause nucleotide composition bias. Furthermore, the efficiency of maximizing protein production by the preferred codons is known as natural selection. Genetic drift results following the eradication of codon changes among generations due to emigration and immigration at the population level [7]. Codon usage patterns can also provide information on the evolutionary process, virus adaptation to the host, genetic drifts, selection, and mutation pressure, among several factors. Variations in gene expression and protein synthesis efficiency may be caused by a bias in the codon usage pattern. In addition, the extent to which the bias in the codon usage pattern affects viral-host adaptiveness influences replication efficiency, virulence, protein synthesis, and virus survival. Several studies have suggested that mutational pressure is the primary driving force behind the formation of a codon use pattern [8, 9].

Bayesian Evolutionary Analysis by Sampling Tree (BEAST) is a quick and easy-to-use software (https://beast.community/) that has become a popular platform for resolving evolutionary analysis and phylogenetic time-trees. It provides the Bayesian Markov Chain Monte Carlo (MCMC) [10] technique or method for phylogenetic reconstruction, which is already the most widely used and fundamental approach. It also creates a platform for analyzing several data partitions simultaneously, which is useful for estimating the single multilocus coalescent analysis. BEAUti is an analysis engine built within the BEAST software that facilitates the creation of a modeling file without any Graphical User Interface (GUI) programming. It offers the ability to check points and restart analysis. Furthermore, it provides a template-based GUI enhancement, an extensible XML format, and a tool tracer, an in-built tool of BEAST software. BEAUti enables the user to see the log file in the graphical format generated after BEAST execution. A tree annotator program is used to burn the tree file. In addition, the Figtree software (https://beast.community/) was used to further visualize the phylogenetic tree and show the year of time of the most recent common ancestor (tMRCA). In contrast, the system requires a Java platform to run the BEAST software in Linux or Ubuntu. To estimate selection and evolutionary pressure on protein-coding areas, the proportion of the substitution rate at non-synonymous and synonymous sites is quantified. The dN/dS ratio is the most used method [9].

The glycoprotein E2 is the major immunodominant glycoprotein on the outer surface of *Pestiviruses*, which induces neutralizing antibodies in the infected host [11, 12]. It plays an important role in virus attachment and entry into the host [13–15], which determines the host specificity of the *Pestiviruses* [16, 17]. Studies have shown that there are no available drugs to treat *Pestiviruses* in animals.

Therefore, this study aimed to investigate the codon usage bias and evolutionary analysis of E2 protein of all *Pestiviruses* affecting animals. This study will provide relevant information to elucidate the processes of gene expression, drug discovery, and epidemiology, which may be useful in designing newer vaccines.

## Materials and Methods

### Ethical approval

Ethical approval was not necessary as the study materials were collected through the public literature database.

### Data collection and sequence editing

The available coding sequences of the E2 gene of *Pestiviruses A*, *B, C, D, F, H*, and *K* were individually downloaded in FASTA format from the GenBank database of National Center for Biotechnology Information (NCBI). MEGA-X software (https://www.megasoftware.net/) and the multiple sequence comparison by log-expectation (MUSCLE) codon were then used to edit and align the coding sequences [18], which were used for further analysis.

### Nucleotide composition analysis

MEGA-X software was used to calculate the overall nucleotide content and frequency of bases at third codon sites (A3, C3, T3, and G3) of *Pestiviruses A, B, C, D, F*, and *H*. In addition, the SeqinR [Biological Sequences Retrieval and Analysis, it is a package of R (https://cran.r-roject.org/ package=seqinr)] was used to obtain the overall frequency of nucleotide bases, including the composition of G+C contents, GC, GC content at the first codon site, GC1, GC content at the second codon site GC2, GC content at the third codon site GC3, and the average of GC contents at first and second codon site GC12 [18].

### Relative dinucleotide abundance analysis

The relative abundance of dinucleotides may play a role in determining codon usage indices. A total of 16 different dinucleotide occurrences are possible. The outline of the dinucleotide abundance frequency defines both mutational and selection pressure. In the present study, the relative dinucleotide abundance in E2 gene of seven *Pestiviruses* was determined using the method established by Karlin and Burge [19] as below

PXY = FXY/(FX FY),

Where FX and FY are the frequencies of individual nucleotides, and dinucleotides are indicated by FXY in the same equation. PXY > 1.23 is regarded as high relative abundance, whereas PXY < 0.78 is considered low relative abundance. The required external library “seqinR” was used to determine the dinucleotide frequencies in the R Studio programming language [8].

### Relative synonymous codon usage (RSCU) analysis

For a specific amino acid, the RSCU method is defined as the ratio of the observed value to the predicted value. The frequency of amino acids or the sequence length does not affect the RSCU values. RSCU estimates above 1.6 values are overrepresented, whereas those with fewer than 0.6 values are underrepresented. However, RSCU estimates between 1.6 and 0.6 are considered unbiased or randomly used. The following formula was used to calculate the RSCU values [20].


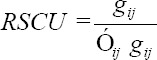


Where *g_ij_* is the observed number of the *i*^th^ codon for the *j*^th^ amino acid, and *ni* is the number of synonymous codons. R Studio programming software (https://cran.r-project.org) and the “seqinR” library were used to obtain and visualize RSCU values for all seven *Pestivirus*es [8].

### The effective number of codons (ENC) analysis

The ENC evaluation reflects the deviation of a codon from random selection. Typically, ENC estimates range from 20 to 60. The value 20 represents an extremely biased situation in which only one codon is used to code for each amino acid. A value of 60 reveals that there is no bias, thus indicating that the codons have been used equally. The codon usage is somewhat biased if the ENC values are fewer than 45. The ENC value was evaluated using the following formula:


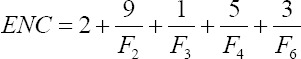


Where *F_i_* (*i* = 2, 3, 4, and 6) denotes the average *F_i_* in the *i* - fold degenerate amino acid family. Where the *Fi* value is calculated using:


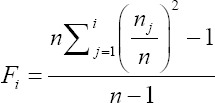


Where *n* is the total number of observed codons for a given amino acid, and *n_j_* is the total number of observed *j_th_* codons for that amino acid. The ENC values for the *Pestiviruses A, B, C, D, F, H*, and *K* were calculated using R Studio programming software using the “vhica [Vertical and Horizontal Inheritance Consistence Analysis, it is package in R. (https://cran.r-project.org/package=vhica)]” library.

The ENC plot was generated to show the relationship between the ENC and GC3 frequencies (the sum of G&C nucleotide at the third codon position). Furthermore, this precise method for estimating absolute synonymous codon usage, defines and quantifies codon usage bias in a gene or genome [8, 21]. The formula for calculating ENC plot values is


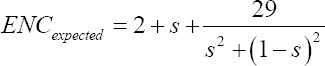


Where *s* indicates the GC3 content. If the ENC values fall on the standard curve, it indicates that mutational pressure is the only factor influencing the codon usage and the values that fall below the standard curve show that the codon bias is constrained by another factor, that is, natural selection [8, 21].

### Neutrality plot analysis

The neutrality plot analysis is used to examine the effect of mutational pressure and natural selection on codon usage patterns. In addition, a neutrality plot was constructed by plotting the GC3 data against the GC12 mean. If the GC3 value is significant and closes to one, mutational pressure plays a major impact in shaping the codon usage pattern over natural selection. However, if the regression slope is = 0, natural selection has a massive impact. A similar technique was used to plot the GC12 values against the GC3 values for each *Pestivirus*. The neutrality plot’s regression line represents the mutational pressure [22, 23].

### Parity rule 2 (PR2) plot analysis

The GC bias on the horizontal axis (G3/[G3+C3]) and AT bias (A3/[A3+T3]) on the vertical axis were plotted in a PR2 or parity rule 2 analysis. Based on the genome makeup, the study generally indicates the relative degree of natural selection and mutation pressure. Both axes will have a 0.5 origin (X = 0.5, Y = 0.5). In addition, the fact that A = T, G = C points are located on the origin implies that natural selection and mutational pressure are not in conflict [22, 24].

### Correspondence analysis (COA)

The codon usage bias varies from one gene to another. Therefore, the COA was used to compute the relationship and variation in codon usage among the *Pestivirus*es *A*, *B*, *C*, *D*, *F*, *H*, and *K*, based on a previous study by Greenacre [25]. The values of 59 synonymous RSCU codons were plotted across two axes (axis-1 and axis-2) in a plot. CodonW (https://codonw.sourceforge.net/) software was used to perform the COA, which was further visualized in R programming software [24].

### Grand average hydrophobicity (GRAVY) and aromaticity (AROMO)

The GRAVY is calculated by dividing the total number of hydropathy values of all amino acids in a sequence by the number of residues. The typical range of hydropathy was between −2.0 and +2.0, where the positive and negative values indicated hydrophobicity and hydrophilicity, respectively. AROMO is the frequency of aromatic amino acids in a sample amino acid sequence, such as Trp, Tyr, and Phe. The total values of GRAVY and AROMO were calculated using the CodonW tool (CodonW download | SourceForge.net) [8].

### Correlation analysis

Correlation analysis was conducted for each *Pestivirus* separately using R Studio programming software and the “corrgram” library. This analysis evaluated the nucleotide composition of A,T,G,C,A3,T3,G3,C3,GC,GC1,GC2,GC3, and other factors such as ENC, CAI, GRAVY, and AROMO [8].

### Evolutionary rate analysis

In this study, complete gene sequences of E2 of all *Pestivirus*es were extracted, aligned, and edited using MEGA-X software. The E2 sequences of *Pestiviruses A*, *B*, *C*, *D*, *F*, *H*, and *K* were from 1992 to 2020, 1990 to 2018, 1966 to 2019, 1994 to 2019, 2010 to 2014, 2014 to 2017, and 2006 to 2019, respectively. Furthermore, a phylogenetic model was selected based on the Akaike information criteria obtained from the jModelTest2 tool. The BEAUti interface of the BEAST software was used to build the input analysis. In addition, the four molecular clock models (relaxed clock log-normal, relaxed clock exponential, strict clock, and random local clock) were considered with Coalescent: Bayesian skyGrid and Coalescent: Extended Bayesian skyline plot trees [7]. Moreover, MCMC methods, a class of algorithms in the BEAST, were used to assess the evolutionary rate and tMRCA. The MCMC chains were repeatedly changed until the constraints had an effective sample extent >200. The BEAST-generated log files were analyzed using the BEAST integrated Tracer tool.

## Results

### Codon usage analysis

#### Data collection and sequence editing

The number of coding sequences of *Pestiviruses A* (n = 89), *B* (n = 60), *C* (n = 75), *D* (n = 10), *F* (n = 07), *H* (n = 52), and *K* (n = 85) was retrieved from the NCBI database. Sequences with 99% similarity were removed from the study. MUSCLE algorithm and MEGA-X software were used to align and edit all protein-coding sequences.

#### Analysis of nucleotide composition and relative dinucleotide abundance frequency

**Figure-1:**
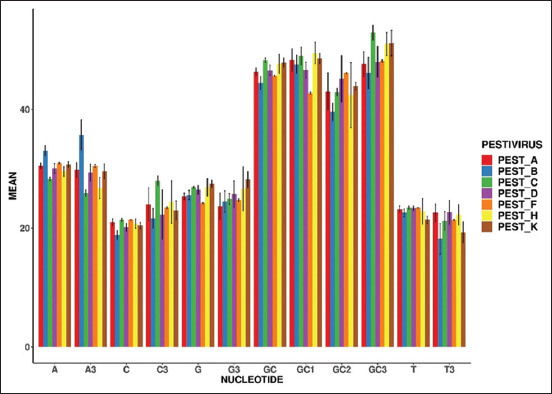
Overall nucleotide composition frequencies (mean) of E2 gene of *Pestiviruses A, B, C, D, F, H*, and *K*.

**Figure-2:**
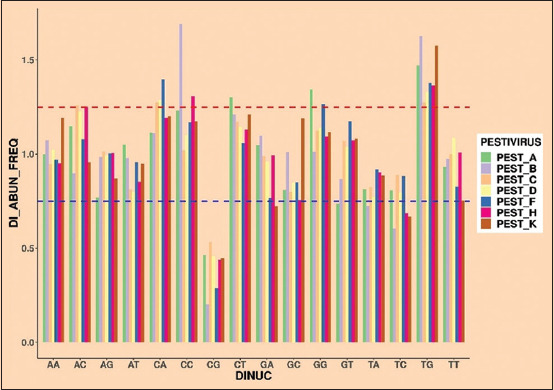
Dinucleotide composition of E2 gene of *Pestiviruses A, B, C, D, F, H*, and *K*.

In this study, the nucleotide composition was analyzed. The evaluated frequency of nucleotide composition is depicted in Table-1 and Figure-1. The findings showed that the nucleotide composition affects the codon usage bias of gene E2 for all *Pestiviruses*. Furthermore, R studio software was used to estimate the relative abundance frequency of 16 dinucleotides of the selected *Pestiviruses*, including *A, B, C, D, F, H*, and *K*. Overrepresented dinucleotides have a frequency value >1.23, whereas underrepresented dinucleotides have a frequency value <0.78. The overall abundance frequency of dinucleotides of all seven *Pestiviruses* is given in Table-2 and Figure-2;

**Table 1:** Nucleotide compositions in E2 gene of *Pestiviruses A, B, C, D, F, H*, and *K*.

Components	A	B	C	D	F	H	K
T	23.21 ± 0.53	22.56 ± 0.68	23.45 ± 0.28	23.35 ± 0.36	23.39 ± 0.07	22.83 ± 2.14	21.41 ± 0.59
C	20.96 ± 0.61	18.83 ± 0.74	21.39 ± 0.30	20.13 ± 0.66	21.42 ± 0.06	20.76 ± 0.79	20.41 ± 0.51
A	30.48 ± 045	33.02 ± 0.89	28.28 ± 0.28	30.05 ± 0.80	30.94 ± 0.11	29.54 ± 0.83	30.70 ± 0.47
G	25.33 ± 0.53	25.57 ± 0.75	26.86 ± 0.17	26.45 ± 0.71	24.23 ± 0.09	26.86 ± 1.48	27.45 ± 0.61
GC	46.30 ± 0.65	44.42 ± 1.08	48.26 ± 0.36	46.58 ± 0.86	45.66 ± 0.07	47.62 ± 1.62	47.87 ± 0.78
GC1	48.28 ± 1.91	47.56 ± 1.56	49.01 ± 1.49	46.62 ± 1.34	42.72 ± 0.15	49.49 ± 1.82	48.57 ± 0.84
GC2	42.98 ± 3.13	39.58 ± 1.50	42.88 ± 0.65	45.15 ± 3.89	46.11 ± 0.10	42.38 ± 5.49	43.90 ± 0.64
GC3	47.61 ± 2.09	46.12 ± 2.65	52.90 ± 1.27	47.98 ± 2.59	48.14 ± 0.20	51.01 ± 1.93	51.15 ± 2.13
T3	22.55 ± 1.51	18.19 ± 2.59	21.22 ± 1.52	22.64 ± 1.99	21.37 ± 0.13	22.25 ± 1.72	19.27 ± 1.76
C3	24.01 ± 2.76	21.66 ± 1.65	27.98 ± 0.78	22.28 ± 4.18	23.42 ± 0.14	24.39 ± 3.56	22.98 ± 1.57
A3	29.76 ± 1.23	35.67 ± 2.54	25.86 ± 0.54	29.37 ± 1.40	30.47 ± 0.24	26.74 ± 1.79	29.56 ± 1.25
G3	23.66 ± 2.25	24.46 ± 1.82	24.92 ± 0.87	25.70 ± 2.24	24.71 ± 0.22	26.60 ± 3.73	28.16 ± 1.34

**Table 2:** Dinucleotide composition of E2 gene of *Pestiviruses A, B, C, D, F, H* and *K.*

Dinucleotides	A	B	C	D	F	H	K
AA	0.9989	1.0723	0.9465	1.0231	0.9703	0.952	1.1916
AC	1.1499	0.8993	1.2584	1.2254	1.0781	1.2518	0.9553
AG	0.7692	0.9848	1.0134	0.9918	1.0033	1.0048	0.8698
AT	1.0514	0.9801	0.8109	0.7982	0.9556	0.8526	0.9481
CA	1.1128	1.1109	1.273	1.2852	1.3975	1.1929	1.1997
CC	1.2301	1.6909	1.0191	1.1017	1.1694	1.3063	1.1743
CG	0.4619	0.2018	0.5327	0.4578	0.289	0.4367	0.4461
CT	1.3014	1.2108	1.1721	1.1428	1.0587	1.1288	1.2091
GA	1.0474	1.0985	0.9896	0.958	0.7673	0.9932	0.7221
GC	0.8083	1.009	0.799	0.8478	0.8501	0.7558	1.1896
GG	1.3449	1.0115	1.1234	1.1382	1.2666	1.0932	1.1168
GT	0.7349	0.867	1.0709	1.0372	1.1737	1.074	1.0812
TA	0.8116	0.7244	0.8244	0.7725	0.9188	0.9036	0.8849
TC	0.8069	0.6054	0.888	0.7942	0.8823	0.6847	0.6682
TG	1.4698	1.6256	1.2734	1.3294	1.3771	1.3642	1.5748

*Pestivirus A*: Among the 16 dinucleotide bases, four were overrepresented >1.23: CC (1.2301), CT (1.3014), GG (1.3449), and TG (1.4698). Likewise, AG (0.7692), CG (0.4619), and GT (0.7349) were found to be underrepresented <0.78.

*Pestivirus B*: Among all the 16 dinucleotide bases, two dinucleotides CC (1.6909) and TG (1.6256) were overrepresented >1.23. Likewise, CG (0.2018), TA (0.7244), and TC (0.6054) were observed as underrepresented <0.78.

*Pestivirus C*: Among all the 16 dinucleotide bases, two dinucleotides AC (1.2582) and TG (1.2734) were overrepresented >1.23. Likewise, CG (0.5327) was observed as underrepresented <0.78.

*Pestivirus D*: Among all the 16 dinucleotide bases, two dinucleotides CA (1.2852) and TG (1.3294) were overrepresented >1.23. Likewise, CG (0.4578) and TA (0.7725) were observed as underrepresented <0.78.

*Pestivirus F*: Among all the 16 dinucleotide bases, three dinucleotides CA (1.3975), GG (1.2666), and TG (1.3771) were overrepresented >1.23. Likewise, CG (0.2890) and GA (0.7673) were observed as underrepresented <0.78.

*Pestivirus H*: Among all the 16 dinucleotide bases, three dinucleotides AC (1.2518), CC (1.3063), and TG (1.3642) were overrepresented >1.23. Likewise, CG (0.4367), GC (0.7558), and TC (0.6847) were observed as underrepresented <0.78.

*Pestivirus K*: Among all the 16 dinucleotide bases, one dinucleotide TG (1.5748) was overrepresented >1.23. Likewise, CG (0.4461), GA (0.7221), TC (0.6682), and TT (0.7544) were observed as underrepresented <0.78.

#### Analysis of RSCU

**Figure-3:**
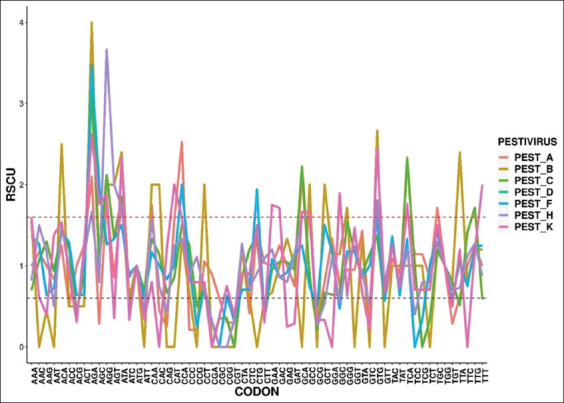
Overall frequencies of relative synonymous codon usage (RSCU) in E2 gene of *Pestiviruses A, B, C, D, F, H*, and *K*.

The RSCU of each *Pestivirus* was determined and plotted using the R studio programming software. Each synonymous codon’s frequency value is classified based on the RSCU, which ranged from 0.6 to 1.6. Values >1.6 were classified as overrepresented synonymous codons, whereas values <0.6 were classified as underrepresented synonymous codons. The over- and under-represented codons are highlighted in yellow and blue, respectively (Table-3). Codons with a significant frequency value >1.0 are referred to as high frequency or positively biased codons. The frequency <1.0, on the other hand, is referred to as a lower frequency or negatively biased codon (Figure-3).

**Table 3:** Relative synonymous codons usage of each amino acid in E2 gene of *Pestiviruses A, B, C, D, F, H*, and *K*. Over represented codons (>1.6) are highlighted in yellow and underrepresented codons (<0.6) are in blue.

Codon	A	B	C	D	F	H	K
AAA	1	1.5556	0.7	1.36	1.3077	0.8182	1.6
AAC	1.1667	0	1.0667	1.2727	1.2308	1.5	0.6154
AAG	1	0.4444	1.3	0.64	0.6923	1.1818	0.4
AAT	0.8333	0	0.9333	0.7273	0.7692	0.5	1.3846
ACA	1.25	2.5	1.4444	1.4194	1.5758	1.5	1.5385
ACC	0.5	0.5	1.2222	1.2903	0.9697	1	0.9231
ACG	1	0.5	0.4444	0.6452	0.3636	0.375	0.3077
ACT	1.25	0.5	0.8889	0.6452	1.0909	1.125	1.2308
AGA	2.1	4	3.1765	3.4737	1.4118	1.6667	2.625
AGC	0.2857	1	1	2	0.6667	0.8	1.7647
AGG	1.8	2	2.1176	1.2632	3.8824	3.6667	1.875
AGT	0.8571	2	1.3333	1.3333	1.1111	2	0.3529
ATA	1.8	2.4	1.8462	1.5	1.9091	1.7143	2.3333
ATC	0.6	0.6	0.4615	0.9	0.6818	0.8571	0.3333
ATG	1	1	1	1	1	1	1
ATT	0.6	0	0.6923	0.6	0.4091	0.4286	0.3333
CAA	1.75	2	1.3333	1.1667	1.0769	1.5714	0.8
CAC	0.4444	2	1.1429	1	1.3333	0.75	0
CAG	0.25	0	0.6667	0.8333	0.9231	0.4286	1.2
CAT	1.5556	0	0.8571	1	0.6667	1.25	2
CCA	2.5263	1.3333	1.5	2	1.92	1.3333	1.6
CCC	0.2105	0.6667	1.25	1	1.12	0.8889	0.8
CCG	0.2105	0	0.5	0.25	0.16	1.1111	0.8
CCT	1.0526	2	0.75	0.75	0.8	0.6667	0.8
CGA	0.9	0	0	0.3158	0	0.3333	0
CGC	0.6	0	0.3529	0	0	0	0.375
CGG	0.3	0	0.3529	0.6316	0.3529	0	0.75
CGT	0.3	0	0	0.3158	0.3529	0.3333	0.375
CTA	1.2414	1.2	0.8571	0.7059	0.8276	1.2727	0.9
CTC	0.4138	0.6	1.2	0.7059	0.6207	0.7273	0.9
CTG	1.4483	0	1.3714	1.9412	1.6552	0.9091	1.5
CTT	1.0345	0.6	0.3429	0.3529	0.8276	1.0909	0.3
GAA	1	0.6667	0.9565	1.0833	1.2	1.2	1.75
GAC	1.25	1	1.0476	0.8571	1.1667	0.8571	1.7143
GAG	1	1.3333	1.0435	0.9167	0.8	0.8	0.25
GAT	0.75	1	0.9524	1.1429	0.8333	1.1429	0.2857
GCA	2.1333	0	2.2222	1.25	1.6	1.6	1.6667
GCC	0.8	2	0.8889	0.75	1.3333	1.6	1.6667
GCG	0.2667	0	0.2222	0.5	0.5333	0.2667	0.3333
GCT	0.8	2	0.6667	1.5	0.5333	0.5333	0.3333
GGA	1.3333	1.1429	0.6452	1.1765	0.6897	1.1034	0
GGC	0.7619	1.1429	0.6452	0.4706	0.6897	0.5517	1.8947
GGG	0.9524	1.7143	1.5484	1.1765	1.5172	1.1034	0.6316
GGT	0.9524	0	1.1613	1.1765	1.1034	1.2414	1.4737
GTA	1.4286	1.3333	0.9143	0.8571	1.0435	0.9032	0.6667
GTC	0.2857	0	1.1429	1	0.8696	0.5161	0.2222
GTG	1.7143	2.6667	1.3714	1.5714	1.913	1.8065	2.4444
GTT	0.5714	0	0.5714	0.5714	0.1739	0.7742	0.6667
TAC	1.0909	1	1.3	1.3636	0.9231	1.2727	1.25
TAT	0.9091	1	0.7	0.6364	1.0769	0.7273	0.75
TCA	1.7143	1	2.3333	1.3333	2.4444	1.2	1.7647
TCC	1.1429	1	1	0	0.4444	0.4	0.7059
TCG	1.1429	1	0	0.3333	0.4444	0.8	0.7059
TCT	0.8571	0	0.3333	1	0.8889	0.8	0.7059
TGC	1.7143	1.2	1.2	1.4118	1.0526	1.2941	1.5
TGG	1	1	1	1	1	1	1
TGT	0.2857	0.8	0.8	0.5882	0.9474	0.7059	0.5
TTA	0.6207	2.4	0.5143	1.0588	0.8276	0.7273	1.2
TTC	1	0.8	1.4118	0.75	1.2727	1.125	0
TTG	1.2414	1.2	1.7143	1.2353	1.2414	1.2727	1.2
TTT	1	1.2	0.5882	1.25	0.7273	0.875	2

*Pestivirus A*: Nine codons were found to be over-represented, while thirteen codons were found to be under-represented. In addition, 26 high-frequency codons and 27 low-frequency codons were discovered.

*Pestivirus B*: Thirteen codons were found to be over-represented, while nineteen codons were found to be under-represented. In addition, 23 high-frequency codons and 28 low-frequency codons were discovered.

*Pestivirus C*: Six codons were found to be over-represented, while 14 codons were found to be under-represented. In addition, 27 high-frequency codons and 31 low-frequency codons were discovered.

*Pestivirus D*: Three codons were found to be over-represented, while 11 codons were found to be under-represented. In addition, 27 high-frequency codons and 28 low-frequency codons were discovered.

*Pestivirus F*: Six codons were found to be over-represented, while 12 codons were found to be under-represented. In addition, 27 high-frequency codons and 34 low-frequency codons were discovered.

*Pestivirus H*: Five codons were found to be over-represented, while 13 codons were found to be under-represented. In addition, 27 high-frequency codons and 31 low-frequency codons were discovered.

*Pestivirus K*: Thirteen codons were found to be over-represented, while 17 codons were found to be under-represented. In addition, 25 high-frequency codons and 33 low-frequency codons were discovered.

#### Analysis of the ENC

**Figure-4:**
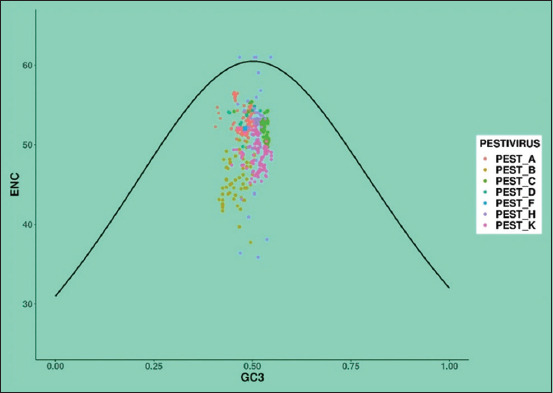
The ENC plot representing the relationship of number of codons against GC3. Each point represents the E2 sequences of *Pestiviruses A, B, C, D, F, H* and *K*.

The ENC was calculated for all of the chosen *Pestiviruses*. Furthermore, the value was used to evaluate the extent of codon usage in a specific *Pestivirus*. However, in this study, the ENC values for *Pestiviruses A, B, C, D, F, H*, and *K* were 48.97–56.59 (standard deviation [SD] ± 1.5848), 37.74–51.50 (SD ± 2.7814), 49.17–55.40 (SD ± 1.3044), 49.78–54.83 (SD ± 1.4489), 51.73–52.20 (SD ± 0.1652), 35.88–61.00 (SD ± 5.6064), and 45.24–52.32 (SD ± 1.8514), respectively. To illustrate and compare the selection and mutational pressure, the ENC of the E2 gene of all *Pestiviruses* was plotted in a single frame (Figure-4). Each color point represents one of the seven *Pestiviruses*. Points located directly below and close to the standard curve indicate a significant role in natural selection and a minor influence from GC3.

#### Analysis of neutrality plot

The nucleotide composition of GC12 (mean value of GC1 and GC2) versus GC3 was calculated to determine the major factors influencing the natural selection and mutational pressure, which were used to plot the neutrality. The slope of the regression line behaves as an indicator. Thus, it is expressed as the evolutionary rate of natural selection and mutational pressure, as shown in the plot. The regression coefficient against GC12 and GC3 is denoted as the natural-mutational equilibrium coefficient. The neutrality plot was interpreted in the following way

**Figure-5:**
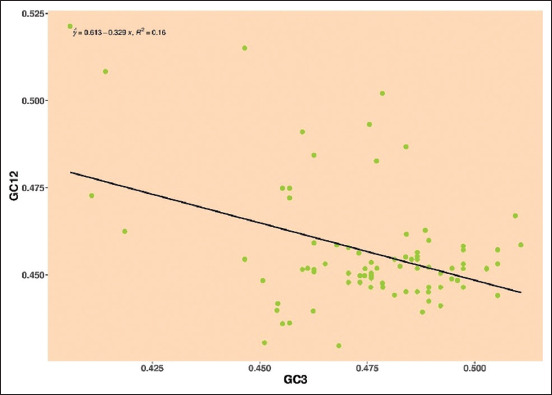
Neutrality plot showing the relationship between GC12% and GC3% with the slope line indicating natural selection. The points represent the E2 sequences of *Pestivirus A*.

*Pestivirus A*: Mean values of GC12 and GC3 were distributed around the regression line, the negative regression line and R-value observed in E2 gene was y = 0.613–0.329, R² = 0.16, that shows 32.9% neutrality indicating the role of natural selection over mutational pressure (Figure-5).

**Figure-6:**
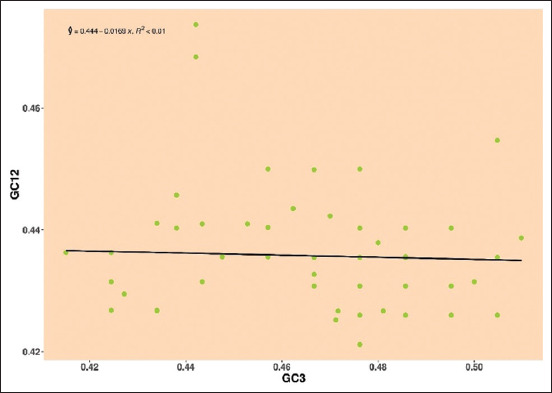
Neutrality plot showing the relationship between GC12% and GC3% with the slope line indicating natural selection. The points represent the E2 sequences of *Pestivirus B*.

*Pestivirus B*: Mean GC12 and GC3 values were enclosed around the regression line, and the negative regression line and R-value were observed in E2 gene was y = 0.444–0.0169, R² < 0.01, and the influence of neutrality was 1.69%, indicating the role of natural selection over mutational pressure (Figure-6).

**Figure-7:**
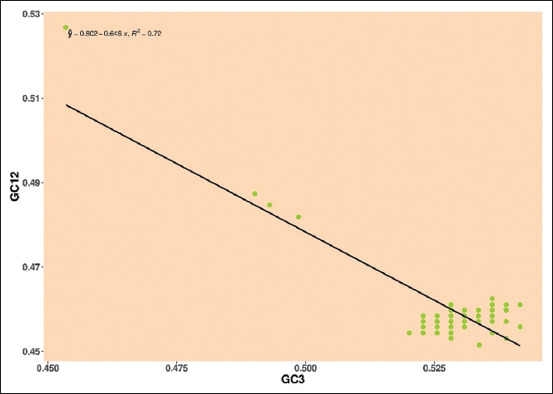
Neutrality plot showing the relationship between GC12% and GC3% with the slope line indicating mutational pressure. The points represent the E2 sequences of *Pestivirus C*.

*Pestivirus C*: Mean values of GC12 and GC3 were surrounded the regression line, the negative regression line and R-value observed in E2 gene was y = 0.802–0.648, R² = 0.72, indicating that neutrality was 64.8%, revealing the role of mutational pressure over natural selection (Figure-7).

**Figure-8:**
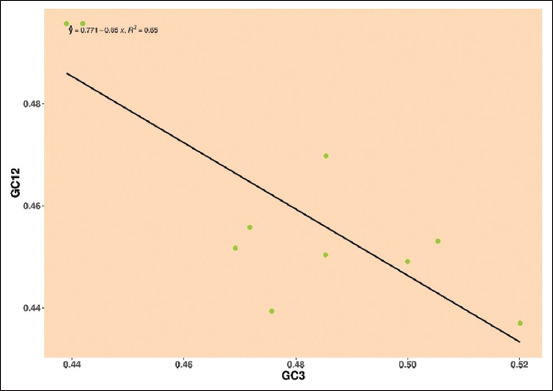
Neutrality plot showing the relationship between GC12% and GC3% with the slope line indicating mutational pressure. The points represent the E2 sequences of *Pestivirus D*.

*Pestivirus D*: The regression line is being surrounded by mean values of GC12 and GC3, and a negative regression line, the negative regression and R-value E2 gene was y = 0.771–0.65, R² = 0.65, indicating the neutrality was 65%, depicting the role of mutational pressure over natural selection (Figure-8).

**Figure-9:**
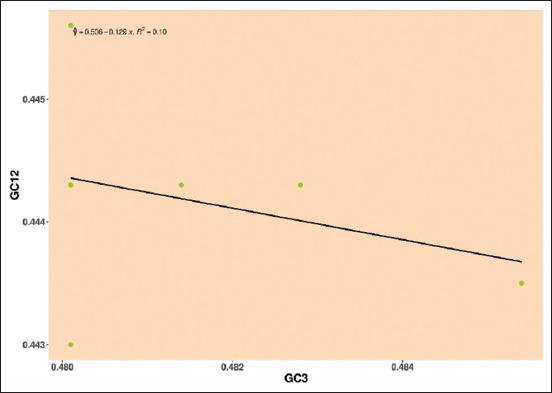
Neutrality plot showing the relationship between GC12% and GC3% with the slope line indicating natural selection. The points represent the E2 sequences of *Pestivirus F*.

*Pestivirus F*: The regression line is being surrounded by mean values of GC12 and GC3, and a negative regression line, the negative regression and R-value E2 gene was y = 0.506–0.129, R² = 0.10, indicating that neutrality is 12.9% revealing the role of natural selection over mutational pressure (Figure-9).

**Figure-10:**
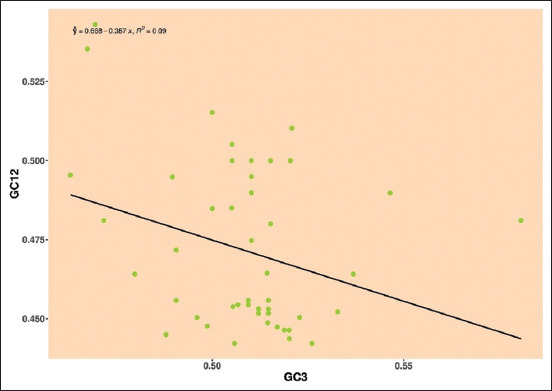
Neutrality plot showing the relationship between GC12% and GC3% with the slope line indicating natural selection. The points represent the E2 sequences of *Pestivirus H*.

*Pestivirus H*: The regression line is being surrounded by mean values of GC12 and GC3, and a negative regression line, the negative regression and R-value E2 gene was y = 0.668–0.387, R² = 0.09, showing that neutrality is 38.7% indicating the role of natural selection over mutational pressure (Figure-10).

**Figure-11:**
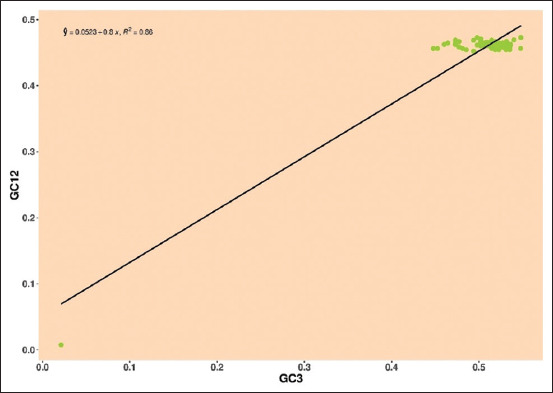
Neutrality plot showing the relationship between GC12% and GC3% with the slope line indicating mutational pressure. The points represent the E2 sequences of *Pestivirus K*.

*Pestivirus K*: The regression line was enclosed by GC12 and GC3 mean values, the positive regression line and R-value in E2 gene was y = 0.0523+0.8, R² = 0.86, showing that neutrality is 80%, indicating the role of mutational pressure over natural selection (Figure-11).

#### Analysis of PR2 plot

**Figure-12:**
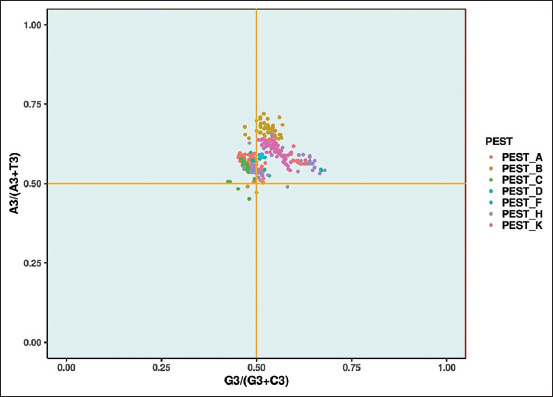
Parity rule 2 plots AT-bias against GC-bias. Each point represents E2 sequences of *Pestiviruses A, B, C, D, F, H*, and *K*.

The direction and degree of the bias are represented by the PR2’s origin. When the PR biases are estimated at the third position of AT and GC content, the PR2 bias plot is found to be comparatively informative. According to Chargaff’s second parity rule, the nucleotide composition of Deoxyribonucleic acid is A=T, G=C. (PR2). Therefore, the origin is the point at which there is no accumulation of bias. The PR2 plot is created by plotting the (G3/[G3+C3]) values on the X-axis against the (A3/[A3+T3]) values on the Y-axis. In this study, the mean values of (G3/[G3+C3)]) and (A3/[A3+T3]) of each *Pestivirus* (Figure-12) are as follows:

1. *Pestivirus A*: The calculated mean value of GC and AT bias was 0.49 and 0.57, respectively, implied AT dominance over the GC.
2. *Pestivirus B*: The calculated mean GC and AT bias values were 0.53 and 0.66, respectively, revealed the domination of AT over the GC.
3. *Pestivirus C*: The calculated mean GC and AT bias values were 0.47 and 0.55, respectively, which indicated AT’s dominance over the GC.
4. *Pestivirus D*: The calculated mean GC and AT bias values were 0.54 and 0.56, respectively, revealed AT dominance over the GC.
5. *Pestivirus F*: The calculated mean GC and AT bias values were 0.51 and 0.58, respectively, indicated AT dominance over the GC.
6. *Pestivirus H*: The calculated mean value of GC and AT bias was 0.55 and 0.56, respectively. Here, Purines and pyrimidines both contribute equally.
7. *Pestivirus K*: The calculated mean GC and AT bias values were 0.55 and 0.60, respectively, revealed AT dominance over the GC.

In this study, none of the genes have an A=T, G=C composition, thereby indicating a bias among the *Pestiviruses* studied. The *A, C, D*, and *H* *Pestiviruses* have a slightly lower bias than those of *B, F*, and *K* *Pestiviruses* because the points of the latter were located away from the origin. Clearly, the PR2 plot indicated that the bias occurred at the third position of AT and GC in the studied *Pestiviruses*, thereby implying that natural selection has a significant influence over mutational pressure.

### COA

**Figure-13:**
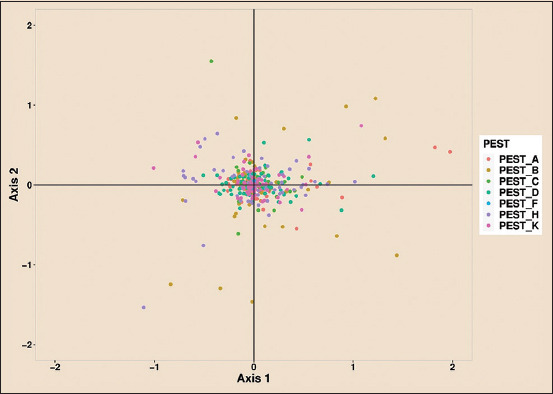
Correspondence analysis, showing a greater contribution of Axis-1 in shaping the codon usage pattern in E2 sequences of *Pestiviruses A, B, C, D, F, H*, and *K*.

In the COA analysis, axis-1 elucidated 9.5%, 33.1%, 13.4%, 5.4%, 0.15%, 1.37%, and 0.65% contribution, whereas axis-2 elucidated 0.69%, 4.9%, 3.2%, 0.85%, 0.05%, 1.0%, and 2.8% contribution for *A, B, C, D, F, H*, and *K Pestiviruses*, respectively. Therefore, axis-1 showed higher usage of the E2 gene of *Pestivirus B* (33.1%) and comparatively lower usage of the E2 gene of other *Pestiviruses* (Figure-13). Axis-1 undertook a different approach when devising the codon usage pattern. Furthermore, the contribution of axis-1 indicated greater codon usage variation among the *Pestiviruses A*, *B*, *C*, *D*, *F*, *H*, and *K*.

#### GRAVY and AROMO

*Pestiviruses A, B, C, D, F, H*, and *K* were evaluated to analyze the correlation of hydrophobicity and AROMO between ENC and GC3. In *Pestivirus* A, ENC-AROMO and ENC-GRAVY had non-significant negative values of −0.32206 and −0.18685, respectively. However, GC3-AROMO and GC3-GRAVY revealed positive but non-significant values of 0.70123 and 0.67793, respectively. In *Pestivirus* B, ENC-AROMO and ENC-GRAVY indicated a negative non-significant value of −0.12289 and a negative significant value of −0.00604, respectively, whereas GC3-AROMO and GC3-GRAVY revealed positive non-significant values of 0.21998 and 0.25379, respectively. In *Pestivirus* C, ENC-AROMO and ENC-GRAVY had negative non-significant values of −0.62046 and −0.42875, respectively, whereas GC3-AROMO and GC3-GRAVY had positive non-significant values of 0.93125 and 0.87876, respectively. In *Pestivirus D*, ENC-AROMO and ENC-GRAVY had non-significant negative values of −0.07384 and −0.34519, respectively, whereas GC3-AROMO and GC3-GRAVY had a negative non-significant value of −0.39537 and a positive non-significant value of 0.76064, respectively. In *Pestivirus F*, all ENC-AROMO, ENC-GRAVY, GC3-AROMO, and GC3-GRAVY values revealed positive non-significant values of 0.22178, 0.64137, 0.76529, and 0.64449, respectively. *In Pestivirus H*, ENC-AROMO and ENC-GRAVY had positive non-significant values of 0.10125 and 0.15759, respectively, whereas GC3-AROMO and GC3-GRAVY had a negative non-significant value of −0.21726 and a positive non-significant value of 0.07654, respectively. *In Pestivirus K*, ENC-AROMO and ENC-GRAVY had positive non-significant values of 0.30111 and 0.13769, respectively, whereas GC3-AROMO and GC3-GRAVY had a positive non-significant value of 0.21688 and a negative non-significant value of −0.18782, respectively. Therefore, the correlation values of GRAVY between ENC and GC3 (hydrophobicity) and AROMO between ENC and GC3 (AROMO) were observed to be non-significant. They were shown to be non-contributory factors while shaping the codon use bias in all seven *Pestiviruses*.

### Evolutionary characteristics analysis

MEGA-X software was used to align and modify the sequences of *Pestiviruses A* (n = 89), *B* (n = 60), *C* (n = 75), *D* (n = 10), *F* (n = 07), *H* (n = 52), and *K* (n = 85).

In addition, the Genetic Algorithm for Recombination Detection was applied to investigate any possible recombination in the *Pestiviruses*. However, the findings showed no evidence of possible recombination in the *Pestiviruses*. Therefore, the dataset comprising the FASTA sequences of the identified genes was directly used to calculate the evolutionary rate.

This was conducted to determine the important changes in the evolutionary rate during the period. Furthermore, by adopting the Bayesian coalescent approach, this study employed complete nucleotide sequences of the E2 gene of *Pestiviruses A*, *B*, *C*, *D*, *F*, *H*, and *K* to calculate the tMRCA and substitution rate (s/s/y). The evolutionary rates for *Pestivirus A, B, C, D, F, H, and K* were calculated as follows 2.67 × 10^−4^ with 95% HPD (lowest 1.36 × 10^−7^, highest 5.9 × 10^−4^), 1.35 × 10^−3^ with 95% HPD (lowest 5.8 × 10^−4^, highest 2.13 × 10^−3^), 6.01 × 10^−4^ with 95% HPD (lowest 3.3 × 10^−4^, highest 9.1 × 10^−4^), 6.53 × 10^−11^ with 95% HPD (lowest 3.3 × 10^−9^, highest 3.5 × 10^−12^), 7.37 × 10^−4^ with 95% HPD (lowest 6.4 × 10^−6^, highest 1.9 × 10^−3^), 1.35 × 10^−3^ with 95% HPD (lowest 3.3 × 10^−8^, highest 3.5 × 10^−3^), and 1.53 × 10^−3^ with 95% HPD (lowest 6.7 × 10^−4^, highest 2.5 × 10^−3^), respectively.

Analyzing the MCC tree of *Pestiviruses A, B, C, D, F, H*, and *K* revealed that the tMRCA ages were 1997, 1975, 1946, 1990, 2004, 1990, and 1990, respectively (Supplementary data can be available from the corresponding author). The evolutionary rate of *Pestivirus C* (53 years) evolved at a rapid rate compared to that of *Pestiviruses A* (28), *B* (28), *D* (25), *F* (04), *H* (13), and *K* (13). Thus, this indicated that *Pestivirus C* was the first virulent virus noted in the *Pestivirus* family.

## Discussion

*Pestiviruses* have shown large genetic diversity. The envelope glycoprotein E2 of *Pestiviruses* *A*, *B*, *C*, *D*, *F*, *H*, and *K* elicits neutralizing antibodies. Therefore, the main target of our study was to assess the codon usage bias and the evolutionary rate of these viruses. The nucleotide composition of each E2 gene in the *Pestivirus* was examined in this study. These findings revealed that nucleotide A was used abundantly in all *Pestiviruses*, which might be a genomic characteristic in members of the genus. Furthermore, the dinucleotides TG were overrepresented among the *Pestiviruses*, except for the *Pestivirus D*, whereas CG was underrepresented in all *Pestiviruses*, thereby indicating that each *Pestivirus* has a varied abundance of dinucleotides. By elucidating the variations or substitutions of the nucleotide compositions, it was shown that the mononucleotide and dinucleotides significantly contribute to shaping the codon usage pattern of the *Pestiviruses*.In addition, the magnitude of the relationship between mutational pressure and natural selection was evaluated for each *Pestivirus*. The PR2 analysis found that the *B*, *F*, *H*, and *K* were more biased compared with *A*, *C*, and *D Pestiviruses*, among the E2 gene of the *Pestiviruses*. According to a previous study on codon usage bias [23], viruses located near the origin of the PR2 plot indicated that the sequence is free of bias.

The average ENC values for the *Pestiviruses* *A*, *B*, *C*, *D*, *F*, *H*, and *K* were 52.95 (SD ± 1.59), 45.62 (SD ± 2.78), 52.0 (SD ± 1.30), 53.42 (SD ± 1.45), 51.97 (SD ± 0.17), 51.57 (SD ± 5.60), and 48.72 (SD ± 1.85), respectively. However, in this study, the ENC scores ranged from 45 to 55, which revealed a moderate level of bias among the sequences, as already demonstrated in the previous studies [5, 22]. Among these *Pestiviruses* of the E2 gene, *Pestivirus* D possessed a higher ENC value, whereas *Pestivirus B* had a lower ENC score. Our findings demonstrated that *Pestiviruses* moderate codon bias may facilitate genome replication and transcription, thereby implying that the virus’s total codon bias is significantly moderate.

The neutrality plot was used to confirm the driving forces of the bias and the number of evolutionary forces. Accordingly, our study’s results showed that natural selection played an important role in *Pestiviruses A*, *B*, *F*, and *H*, whereas mutational pressure influenced the *Pestiviruses C*, *D*, and *K*.

In addition, this study assessed the physical features of amino acids, including hydrophobicity and AROMO and the correlation for all seven *Pestiviruses*. Findings showed that the hydrophobicity and AROMO of codons did not affect the pattern of codon usage bias. Therefore, the results were found to be insignificant for each of the seven *Pestiviruses*.

The most recent common ancestor of lineages is 1997, 1975, 1946, 1990, 2004, 1990, and 1990 for *Pestiviruses A*, *B*, *C*, *D*, *F*, *H*, and *K*, respectively. Based on our observations, this study confirmed the significant role of mutational pressure and natural selection in shaping the codon use bias and evolutionary studies. Thus far, this is the first report of such a study.

## Conclusion

The glycoprotein E2 of all *Pestivirus* is a potent protein that induces high neutralizing antibodies in the host. The E2 gene has been extensively used in phylogeny to subgroup virus isolates. Glycoprotein E2 is a potent subunit vaccine candidate. However, the *Pestivirus*, namely, BVDV-1, BVDV-2, CSFV, and to a certain extent BDV, have been studied extensively concerning molecular epidemiology/phylogeny compared with the other *pestivirus*es. The *Pestivirus*-infected animals have always elicited an immune response majorly to E2 protein, and thus the E2 gene has been evolving over some time. The mutational and selection factors under the immune response, in addition to compositional considerations, were found to play a substantial role in shaping the codon usage pattern. Analyzing the codon usage pattern can help with protein and gene expression optimization analysis. In addition, it facilitates the development of alternative viral vector vaccine options. Similarly, codon usage information can also be used to inhibit viral protein synthesis during replication, in contrast to enhancing the protein expression. This study may also provide information on codon usage for other viruses; thus, investigations can be conducted to explore other applications as well.

## Authors’ Contributions

MS: Analyzed the data and prepared the results. KPS: Guided and supervised in every step of the work. UBI: Prepared tables and graphs. MSB: Prepared tables and edited the manuscript. NM: Collected the data, conducted the analysis and drafted the manuscript. CS: Analyzed the data. RRA: Drafted the manuscript. ES: Analyzed the data and drafted and edited the manuscript. VS: Extracted the data and edited the manuscript. SPK: Extraction and documentation of data. BRS: Reviewed and edited the manuscript. SKP: Prepared the graphs, and edited the manuscript. SSP: Extracted the data, drafted, and edited the manuscript. All authors have read and approved the manuscript.
